# Status of self-esteem in medical students at a college in Kathmandu: A descriptive cross-sectional study

**DOI:** 10.12688/f1000research.72824.2

**Published:** 2022-04-11

**Authors:** Bikal Shrestha, Stuti Yadav, Subodh Dhakal, Pooja Ghimire, Yubika Shrestha, Ela Singh Rathaure

**Affiliations:** 1 Nepalese Army Institute of Health Sciences (NAIHS), Kathmandu, 44600, Nepal; 2 Epidemiology and Disease control division, USAID, Kathmandu, 44600, Nepal

**Keywords:** self-esteem, college, medical students, Kathmandu, Nepal, undergraduates, doctors, self-perception

## Abstract

**Background:** Self-esteem is vital to living a happy, confident and content life. Medical students experience various forms of stress due to academic, financial and social pressures which could affect their levels of self-esteem. This study aims to study the status of self-esteem among undergraduates of a medical college at Tribhuvan University, Nepal.

**Methods:** After receiving the ethical approval from the Institutional Review Committee (IRC) of NAIHS; we conducted a descriptive cross-sectional study among the first- to fifth-year medical students from December 2020 to April 2021. 190 were selected for the study using a stratified random sampling technique. This study used the Rosenberg self-esteem scale to measure self–esteem of the participants. A Google Forms questionnaire was sent to the participants via email. Then, the data obtained were entered in the Google sheet and later analyzed using SPSS 27. A Chi-square test was used to identify potential differences in self-esteem scores among different variables. A p-value of < 0.05 was considered statistically significant.

**Results:** This study included a total of 180 participants, among which, 18.9% (13.19% to 24.61%; at 95% CI) students showed low self -esteem. 74.4% (68.02% to 80.78%; at 95% CI) students had normal self-esteem and 6.7% (3.05% to 10.35%; at 95% CI) students had high self-esteem. The mean self-esteem score was 19.19 (15.01 to 23.37; at 95% CI). Female participants suffered more than males from low self-esteem, and third-year students had the highest percentage of low self-esteem (30.77%).

**Conclusion:** The majority (74.4%) of medical students had normal self-esteem. However, 18.9% students had low self-esteem, among which, third-year students suffered the most (30.77%). Likewise, females exhibited higher prevalence of low self-esteem compared to males. Interventions to boost the level of self-esteem should be carried out to help medical students become confident and efficient doctors.

## Introduction

Self-esteem is one’s sense of self-worth, acceptance, and confidence that individuals feel toward themselves. ^1^ Mentally and physically active people are the foundation of a healthy society and serve the community. Doctors and medical students in particular must have a healthy mental state. A doctor suffering from anxiety, dissatisfaction, and low self-esteem will not be able to fulfil his/her duty properly, ^2^ and a medical student with low self-esteem will never grow to become a healthy, efficient doctor.

Having balanced self-esteem is highly important for healthcare professionals, including medical students. ^3^ Medical students undergo major stress in the form of academic workload, ^4^ family expectations, peer pressure, financial issues ^5^^,^^6^ and excessive working hours. ^5^^-^^7^ Studies show that a large proportion of medical students are unhappy and have low self-esteem, ^8^ and that students with low self-esteem are likely to suffer from anxiety and mental breakdowns, doubt themselves and feel a sense of inadequacy. ^9^ These students tend to appear less competent among their peers ^10^ which contributes to depression and unhappiness. Self-development and well-being is also negatively affected by inadequate self-esteem. ^8^

Self-esteem influences a student's performance, as well as their physical and psychological health and therefore is important to be maintained. A person who has high self-esteem can cope more effectively with stress and anxiety-inducing events in life without any negativity or impact on their mental health. ^11^ It helps one to foster confidence, appear competent among their colleagues, tackle challenges with optimism and take on criticism without it affecting their self-image. ^8^

In Nepal, studies evaluating the self-esteem of medical students are lacking, but existing literature studying nursing students found that nearly 78% of the students had low self-esteem. ^12^ This study uses Rosenberg Self Esteem Scale (RSES). It is a widely and commonly used scale for measuring self-esteem. ^12^^,^^13^ It classifies self-esteem as high, normal and low self esteem based on the scores obtained. ^13^

Assessing medical students’ self-esteem and designing necessary interventions for students with inadequate self-esteem can help us produce confident and optimistic doctors. Since confidence is regarded as a subjective marker of competence, ^14^ we can expect to produce highly competent doctors from the timely intervention. Having this competency in student life will help them acquire good knowledge and later, as a doctor, enable them to effectively manage patients. This study aims to report the status of self-esteem among medical students at a college in Kathmandu.

## Methods

### Study design and setting

A descriptive cross-sectional study was conducted from December 2020 to April 2021. Data collection took place from 30 ^th^ of December, 2020 to 25 ^th^ of January, 2021 among medical students at the Nepalese Army Institute of Health Sciences-College of Medicine (NAIHS-COM) situated in Kathmandu. Medical undergraduates from all years of study were included in the study. The study was conducted after receiving ethical approval from the Institutional Review Committee (IRC), NAIHS; on December 2020 (Reg. No. 373).

### Sample size

We calculated the adequate sample size using the formula for infinite population and then adjusted it according to our population size. Based on Syed *et al*’s study, we estimated the proportion of students with low self-esteem to be 18%. ^15^ Using this estimation, we calculated the minimum sample size that could represent our population.

N=z^2×p×1−p/d2

[where, N = sample size for infinite population, z = factor to achieve (1 − a) % level of confidence (z = 1.96 at 95% C.I) p = estimated rate of population proportion i.e. 18% here. ^15^ d = margin of error to be tolerated (5% here)]

N=1.96^2×0.18×1−0.18/0.05^2

N=226.80i.e.227

For our study population, adjusted sample size is:

$$n=N/\left(1+\left(N-1/P\right)\right)$$

[where, n = adjusted sample size for our finite population, P = our study population i.e. 550 students.]

$$n=227/\left(1+226/550\right)=160.89\;i.e.161$$

Hence, our sample size (n) is 161.

Adjusting the sample size by taking non-response rate at 15%, the final sample size becomes 190.

### Sampling strategy and eligibility criteria

Students 18 years or older who gave consent were eligible to participate in the study. For the purpose of sampling, students were divided into two strata: pre-clinical (first, second) and clinical year (third, fourth, fifth). This was done because students of pre-clinical years shared some common characteristics – such as studying basic medical subjects without clinical exposure, while students of clinical years had clinical exposure. Students were selected through a Proportionate Stratified Random sampling technique so that every student from the first to fifth year had an equal probability of being selected in the study. This would ensure that the sample within each stratum properly represented our study population. The sampling frame was prepared by collecting the list of students from administrative section of the institute, who were numbered from 1 to 550. Within that limit, 190 random numbers were generated with the help of Google Random Number Generator as per the proportion of students from each year and the proportion of males and females in each year. The random numbers that would represent the student researchers involved in this study were excluded.

### Survey instrument

To measure self-esteem, we used a questionnaire based on the Rosenberg Self Esteem Scale (RSES). ^13^^,^^16^^,^^17^ The questionnaire had two parts; demographic details and the Rosenberg Self Esteem Scale. Demographic details covered general information including: age, sex, province of permanent address, year of study and type of institution they had attended for primary level education. A copy of the questionnaire can be found in the extended data. ^18^

The RSES was developed by sociologist Morris Rosenberg. ^16^ It is a 10-item Likert-type scale with items answered on a four-point scale from strongly agree to strongly disagree. For items 1, 3, 4, 7, 10: Strongly Agree = 3, Agree = 2, Disagree = 1, and Strongly Disagree = 0. For items 2, 5, 6, 8, 9 (which are negative statements; hence reversed in score): Strongly Agree = 0, Agree = 1, Disagree = 2, and Strongly Disagree = 3. ^16^^,^^18^ A total is obtained by adding these markings which ranges from 0–30. A score less than 16 indicates low self-esteem, more than 25 indicated high self-esteem and scores from 16 to 25 shows normal self-esteem in the respondents. ^13^^,^^17^

The RSES has high reliability: test-retest correlations are in the range of .82 to .88 and Cronbach's alpha for various samples are in the range of  .77 to  .88. ^19^^,^^20^ We also calculated Cronbach’s alpha for our study sample and the value for it was 0.811 which showed good internal consistency and reliability of the instrument for our population as well.

### Data collection and statistical analysis

The questionnaire was sent to 190 students through Google forms via email. Out of 190 students, 182 responded among which 2 refused to give consent. Hence, we obtained complete data from 180 students which was then entered into a Google spreadsheet ^18^ and later analyzed using Microsoft Excel and SPSS version 27. Chi Square Test of Association was used to compare between and among different categorical variables by taking the median values of RSES score. A p-value of < 0.05 was considered statistically significant.

### Potential biases

The participant responses could have been subjected to response bias and subjective bias. Response bias includes responding to questionnaire items without understanding them and also giving socially desirable answers. Subjective bias could have occurred as the questionnaire is based on the subjective feelings of the respondents. We could not control subjective bias as it is inevitable when measuring abstract parameters such as self-esteem. But for response bias, we employed certain techniques to minimize it as far as possible. The questionnaire was self-administered, without individual interpretation by the researcher, but with explanation for some possible ambiguities in it. We checked the clarity of the items by translating the items to Nepali and retranslating it back to English with the help of two individuals who were not involved in the study. Moreover, we assured participants regarding concealed identity and confidentiality issues at the beginning and the Likert scale used was a 4-point Likert without the neutral responding.

### Ethical statement

Ethical approval was obtained from the Institutional Review Committee (IRC), NAIHS in December 2020 (Reg. No. 373). There was no patient or public involvement in the design, conduct or reporting of our research. The first section of our Google forms survey had an information sheet with information about our study and an informed consent section where the participants could choose to agree or disagree to participate in the study via a tick-box. Participants who chose to agree could proceed to the questionnaire section. They were informed that their identity would be concealed and their de-identified data would be accessed only by the researchers. In this way, only after obtaining informed consent, the randomly selected medical students were involved as participants for data collection.

## Results

180 responses were received from 190 study participants (94.73% response rate). Out of the 180 participants, 123 (68.3%) were male and 57 (31.7%) were female. Majority of the respondents were from clinical years (third, fourth and fifth year) making up 63.5%, whereas only 36.1% respondents were from Pre-Clinical years (first and second year). The average age of the students was 22.17 ± 1.78 years. The mean self-esteem score of the respondents was 19.19 ± 4.18 (15.01 to 23.37 at 95% CI) which lies in the normal range of RSES score.

Among 180 participants, 18.9% (13.19 to 24.61 at 95% CI) were found to have low self-esteem. 74.4% (68.02% to 80.78% at 95% CI) students had normal self-esteem and 6.7% (3.05% to 10.35% at 95% CI) students had high self-esteem.

Female participants were found to have suffered lower self-esteem than males. The mean self-esteem score of males was 19.63 ± 4.29 and that of females was 18.26 ± 3.81. There is an association between sex and median values of RSES scores (i.e., whether students had higher or lower self-esteem than the median value). Significant difference in self-esteem was found between males and females at 5% level of significance (χ ^2^ = 4.33, p = 0.037). Not enough evidence was found to suggest a significant difference in RSES scores between and among any other variables. Out of 65 pre-clinical year students, 11 (16.92%) and out of 115 clinical year students 23 (20%) were found to have low self-esteem. Third year medical students had the highest percentage of low self-esteem i.e., 12 (30.77%) as shown in Table 1.

**Table 1.** Socio-demographic characteristics and RSES of the students along with p- value.

Variables		RSES SCORE			Total	p-value
		< 16	16 – 25	>25		
**Sex**	Male	22 (17.89%)	91 (73.98%)	10 (8.13%)	123	0.037
	Female	12 (21.05%)	43 (75.44%)	2 (3.51%)	57	
**Type of primary schooling**	Government	2 (10%)	16 (80%)	2 (10%)	20	0.508
	Private	32 (20%)	118 (73.75%)	10 (6.25%)	160	
**Year of medical college**	First year	6 (17.65%)	26 (76.47%)	2 (5.88%)	34	0.660
	Second year	5 (16.13%)	23 (74.19%)	3 (9.68%)	31	
	Third year	12 (30.77%)	25 (64.10%)	2 (5.13%)	39	
	Fourth year	5 (14.71%)	28 (82.35%)	1 (2.94%)	34	
	Fifth year	6 (14.29%)	32 (76.19%)	4 (9.52%)	42	
**Level of education**	Pre-clinical	11 (16.92%)	49 (75.38%)	5 (7.69%)	65	0.972
	Clinical	23 (20%)	85 (73.91%)	7 (6.09%)	115	
**Province**	Province No. 1	1 (9.09%)	9 (81.82%)	1 (9.09%)	11	0.939
	Province No. 2	9 (26.47%)	23 (67.65%)	2 (5.88%)	34	
	Bagmati Province	13 (16.25%)	61 (76.25%)	6 (7.5%)	80	
	Gandaki Province	5 (20.83%)	18 (75.00%)	1 (4.17%)	24	
	Lumbini Province	5 (21.74%)	16 (69.57%)	2 (8.7	23	
	Karnali Province	0 (0%)	3 (100%)	0 (0%)	3	
	Sudurpashchim Province	1 (20.00%)	4 (80.00%)	0 (0%)	5	

It was found that 20% (14.16% to 25.84% at 95% CI) of the students who had attended private school for their primary level of schooling had low self-esteem whereas its prevalence among government school students was reported to be 10% (5.62% to 14.38% at 95% CI). Further details of the findings have been presented in Table 1.

## Discussion

Self-esteem refers to an individual’s overall sense of value or worth. ^19^ Various factors including age, ^21^ sex, ^22^^,^^23^ socio-economic status, ^23^^,^^24^ level of study, ^25^ academic stress ^12^ and family environment ^26^ influence self-esteem. A reasonably high degree of self-esteem is considered an attribute of good mental health, ^27^ and students with high self-esteem are found to have better academic achievements compared to those with low self-esteem. ^28^ So, in order to improve the academic performance of medical students, healthy self-esteem is imperative. Moreover, healthcare professionals with high self-esteem show higher tendency to influence and induce positive well-being not only in patients but also in the healthcare team. ^29^ Empathy is an essential element of the physician-patient relationship and is also an essential quality for medical students. ^30^ A medical student with low self-esteem is likely to grow less empathetic since self-esteem and empathy are positively correlated. ^30^ Low self-esteem has also been allied with unhappiness, ^31^ suicidal ideation, ^32^ isolation and avoidance of social settings ^33^ and psychological disorders. ^34^

Numerous studies have been conducted on self-esteem among the general population but there have been limited studies conducted among medical students. According to studies carried out by Syed, *et al*. and Nair, *et al*. among medical students, the prevalence of low self-esteem was found to be 18% ^15^ and 19.4% ^35^ respectively. According to our study, the prevalence of low self-esteem among the medical students of NAIHS-COM was found to be 18.9% which was similar to the above mentioned studies. This prevalence of low self-esteem may be due to high academic stress, ^12^ low perceived family support ^12^ and learning environment. ^24^ Since not many studies have been conducted on self-esteem among medical students in Nepal, other contributing factors are yet to be explored. 74.4% of the students had normal levels of self-esteem. Having high but realistic self-esteem is important for sound mental health. But considering the potential for subjective bias in this study, it cannot be implied that the high scores (>25) attained by the respondents are an accurate reflection of their actual self-esteem status, and not a subjective, narcissistic trait. Narcissistic people are self-centered people who do not care much about others. The prevalence of high self-esteem was reported to be 6.7% in our study. Studies among Arab participants suggest that self-esteem positively and significantly linked with attitude towards life, mental health, and general satisfaction. ^36^^-^^41^ People with high self-esteem are expected to persist in the face of challenges than those with low self-esteem. ^42^ But it is also suggested that people with high self-esteem are more likely to be conceited, arrogant and sometimes narcissistic. ^43^

The mean RSES score of males was found to be significantly higher at 19.63 ± 4.29, whereas females had a score of 18.26 ± 3.8. Studies conducted among Malaysian and Indian students showed no significant difference between males and females. ^44^^,^^45^ A study done among four Arab counties showed Kuwaiti men had a significantly higher mean score than their female peers. ^40^ A study conducted in Loni, India also showed higher prevalence of low self-esteem in males than females, ^15^ whereas our study indicated that low self-esteem was more prevalent among females.

In our study, significant differences between self-esteem were not found among clinical and pre-clinical year students. However, third year medical students had the highest prevalence of low self-esteem. This could be due to lack of adaptation to academic burden while transitioning from pre-clinical phase to clinical phase. A study conducted in India by Syed *et al* had a dissimilar finding that the cohort of first year males had the highest prevalence of low self-esteem. ^15^ In a study conducted by Naz, *et al*, the highest prevalence of low self-esteem was found in fourth year medical students. ^46^ As the third year medical students had the highest prevalence of low self-esteem, proper orientation and pre-exposure sessions should be conducted before entering the clinical phase. Psychological counselling should be frequently provided to all medical students, focusing more on the students with low self-esteem. Medical students should also be encouraged to participate in extracurricular activities which may help in boosting their self-esteem. This is because involvement in extracurricular activities can help students achieve personal identity development and a sense of self-worth ^47^ which will in turn, add to their self-esteem.

A Nigerian study showed that self-esteem is positively and significantly correlated with the primary school attended. ^24^ The self-esteem of medical students who attended private schools was significantly higher than those of public school students. ^24^^,^^48^ In our study, we tried to figure out whether there was any difference in self-esteem between students who had attended government schools for primary level of schooling and those who had attended private schools. Although we found that a proportionately higher number of students who had acquired primary levels of education through private schools had low self-esteem, the difference was not significant. We propose that this could be because the majority of the students (88.9%) were from private schools and a comparatively higher percentage of low self-esteem was seen among them. But the statistical significance of this difference was not obtained which could have been due to the very low number of students from government schools for the comparison.

We were also interested to know if the permanent address of students created an impact on their self-esteem since not all parts of Nepal are equally developed and students from some areas may be deprived of opportunities. But we did not find any significant difference in the self-esteem of students from different provinces.

Self-esteem can be classified as explicit and implicit self-esteem. ^49^ Explicit self-esteem is presented in the reflective and deliberative evaluations that one makes about oneself, while implicit self-esteem results from responses to self-relevant stimuli and is the function of automatic process. ^50^ It is found that people with high explicit self-esteem and low implicit self-esteem showed higher levels of narcissism ^49^ and the self-esteem level measured with RSES is usually the explicit one. ^51^ So, a self-esteem score of more than 25 cannot always be guaranteed to be healthy since high self-esteem can be relatively secure or defensive. ^49^ Our study showed that 6.7% students had high self-esteem. The normal range of self-esteem falls within the 16-25 score range which 74.4% of the students come under.

Healthy self-esteem in healthcare professionals is a personal quality which, when combined with professionalism and accountability, can greatly reinforce patient satisfaction. ^52^ This is the reason why strengthening medical students’ self-esteem warrants special consideration from the beginning of their medical studies as early intervention could yield better results.

### Strengths and limitations

The findings of this study contribute evidence to the limited literature on the status of self-esteem in medical undergraduates at a college in Nepal. Our findings provide estimations of our future doctors’ levels of self-esteem. This would help the policy makers and medical institution to develop interventions that could support those with low self-esteem. The study used well-known standard screening tools i.e. RSES to assess self-esteem and measured its difference among several variables. The RSES has high reliability. ^19^^,^^20^ We also calculated Cronbach’s alpha for our study sample and the value for it was 0.811 which showed good internal consistency of the instrument for our population as well.

As the study was conducted among medical students at a single medical college, the results cannot be generalized to the larger populations of the students of other medical colleges of Nepal as well as the general population. Therefore, multi-centric studies on self-esteem should be conducted in order to expand the generalizability of the findings. Likewise, Rosenberg’s self-esteem scale only measures general self-esteem neglecting self-esteem in relation to peers, parents and schooling. ^53^ This leads to partial assessment of levels of self-esteem. The participants may have felt pressure to give answers that were socially desirable which could have contributed to response bias. Response bias includes responding to questionnaire items without understanding them and also, providing what they consider to be socially desirable answers. There might also be subjective bias associated with our study as the questionnaire is based on the subjective feelings of the respondents.

## Conclusion

This study found that the majority of medical students had normal self-esteem. Several factors including age, gender, schooling, and year of study could have an impact upon self-esteem. Further studies on self-esteem should be conducted on larger populations to determine the contributing factors of low self-esteem in medical students. In our study, low self-esteem was most prevalent in third year students which may be due to the transition from pre-clinical to clinical study. Proper counseling and clinical exposure in pre-clinical years may help students to manage this transition more easily. Considering the conducted study and the related studies, we can conclude that self-esteem along with medical knowledge and skills play a great role in shaping future doctors. Hence, it is recommended that essential measures such as clinical exposure in pre-clinical years and proper counseling should be used to enhance the levels of self-esteem and mental health among medical students.

## Conflict of interest

No competing interests were disclosed.

## Funding

The author(s) declared that no grants were involved in supporting this work.

## Data availability

### Underlying data

Figshare: ‘Status of self-esteem in medical students of a college in Kathmandu: A descriptive cross sectional study’. https://doi.org/10.6084/m9.figshare.15149589. ^18^

### Extended data

Figshare: ‘Status of self-esteem in medical students of a college in Kathmandu: A descriptive cross sectional study’. https://doi.org/10.6084/m9.figshare.15149589. ^18^

This project contains the following extended data:

- Questionnaire (includes consent form)

### Reporting guidelines

STROBE checklist for ‘Status of self-esteem in medical students of a college in Kathmandu: A descriptive cross sectional study’, https://doi.org/10.6084/m9.figshare.15149589. ^18^

Data are available under the terms of the Creative Commons Attribution 4.0 International license (CC-BY 4.0).
