# Supplementary material for: Status of self-esteem in medical students at a college in Kathmandu: A descriptive cross-sectional study
Source: F1000Res. 2022 Apr 11;10:1031. Originally published 2021 Oct 11. [Version 2] doi: 10.12688/f1000research.72824.2 (PMC9021663; doi:10.12688/f1000research.72824.2)
Supplement: Peer review history

# Peer review: Reviewer response for version 2

**DOI:** 10.5256/f1000research.124413.r134526

**Published:** 2022-04-11

**Authors:** Sunil Kumar Joshi

**Affiliations:** 1 Nepal Injury Research Centre, Department of Community Medicine, Kathmandu Medical College, Kathmandu, Nepal

After the revision of the manuscript, I accept the submission.

Is the work clearly and accurately presented and does it cite the current literature?

Partly

If applicable, is the statistical analysis and its interpretation appropriate?

No

Are all the source data underlying the results available to ensure full reproducibility?

Yes

Is the study design appropriate and is the work technically sound?

Yes

Are the conclusions drawn adequately supported by the results?

Yes

Are sufficient details of methods and analysis provided to allow replication by others?

Partly

Reviewer Expertise:

Public health, Occupational health and safety, injury and violence prevention.

I confirm that I have read this submission and believe that I have an appropriate level of expertise to confirm that it is of an acceptable scientific standard.

# Peer review: Reviewer response for version 1

**DOI:** 10.5256/f1000research.76429.r123050

**Published:** 2022-03-09

**Authors:** Sunil Kumar Joshi

**Affiliations:** 1 Nepal Injury Research Centre, Department of Community Medicine, Kathmandu Medical College, Kathmandu, Nepal

I appreciate the efforts put forward by a group of young students to participate in such a novel study in Nepal. This study on self-esteem has been conducted in a single medical college in Kathmandu.

 Here are a few comments from me:

 There is no explanation on the classification of self-esteem and its relation to the RSES scoring in the Introduction section, it comes for the first time in the Methods section.

 Few sentences need to be rephrased or restructured as they are not very clear to me. The manuscript needs to be checked by an English language expert. The authors should elaborate on the technical words mentioned in the text, e.g., narcissism.

 In the pdf format of the manuscript "Table 1. Socio-demographic characteristics and RSES of the students along with p-value" comes in the Methods section, it should go to the Results section.

 I have some reservations about the sample size calculation. An estimated rate of population proportion from a study in a rural Indian medical college i.e.18% has been cited to calculate the sample size (ref 15). NAIHS is not a rural medical college, and as it is the first of its kind study in Nepal, I suggest using the population proportion of 50%. It would bring a sample size of approximately 384. I do not support the adjusted sample size calculation for the finite population in this study. As the total number of students in this medical college is 550, the researchers would be easily able to get the required study respondents. So, I suggest the students do recalculate the sample size as I suggested, extend the data collection and reanalyse data and resubmit for publication. This will definitely make the findings more robust. I suggest being considerate in citing the published literature. I think a total of 53 references is a little high for this article.

Is the work clearly and accurately presented and does it cite the current literature?

Partly

If applicable, is the statistical analysis and its interpretation appropriate?

No

Are all the source data underlying the results available to ensure full reproducibility?

Yes

Is the study design appropriate and is the work technically sound?

Yes

Are the conclusions drawn adequately supported by the results?

Yes

Are sufficient details of methods and analysis provided to allow replication by others?

Partly

Reviewer Expertise:

Public health, Occupational health and safety, injury and violence prevention.

I confirm that I have read this submission and believe that I have an appropriate level of expertise to state that I do not consider it to be of an acceptable scientific standard, for reasons outlined above.

# Response

**Published:** 2022-04-05

**Authors:** Stuti Yadav

**Affiliations:** Nepalese Army Institute Of Health Sciences, Nepal

Thank you for the comments, sir. The manuscript has been changed as per the comments and the table has also been placed in the results section.

# Peer review: Reviewer response for version 1

**DOI:** 10.5256/f1000research.76429.r98205

**Published:** 2022-02-03

**Authors:** Christian Sunday Ugwuanyi

**Affiliations:** 1 Department of Science Education, Faculty of Education, University of Nigeria, Nsukka, Nigeria

The study explored the state of self-esteem among undergraduates at Tribhuvan University in Nepal's medical college. The majority of medical students reported normal self-esteem, according to the findings of a descriptive cross-sectional survey conducted among first- to fifth-year medical students from December 2020 to April 2021. The findings, however, revealed that 18.9% of students showed low self-esteem, with third-year students being the most affected (30.77 percent). The researchers recommended that to help medical students become confident and effective doctors, interventions to improve self-esteem should be implemented. This study was properly conceptualised and documented as an academic paper, but more efforts should be made by the authors to use recent empirical studies to support their line of argument. The methodology section of the manuscript was properly handled to allow for its replication. In other words, sufficient details of methods and analysis were provided to allow replication by others. The results were also adequately presented.

Is the work clearly and accurately presented and does it cite the current literature?

Partly

If applicable, is the statistical analysis and its interpretation appropriate?

Yes

Are all the source data underlying the results available to ensure full reproducibility?

Yes

Is the study design appropriate and is the work technically sound?

Yes

Are the conclusions drawn adequately supported by the results?

Yes

Are sufficient details of methods and analysis provided to allow replication by others?

Yes

Reviewer Expertise:

Educational research, measurement and evaluation

I confirm that I have read this submission and believe that I have an appropriate level of expertise to confirm that it is of an acceptable scientific standard, however I have significant reservations, as outlined above.

# Peer review: Reviewer response for version 1

**DOI:** 10.5256/f1000research.76429.r100126

**Published:** 2021-11-22

**Authors:** Dr. Richard Mottershead

**Affiliations:** 1 Ras Al Khaimah College of Nursing, RAK Medical and Health Sciences University, Ras Al Khaimah, United Arab Emirates

I would like to say thank you for asking me to review this research. I have read the article and have the following comments to make:

 This is a generally well-composed paper, which will be of interest to healthcare educators who wish to be aware of issues which may hamper student performance and well-being. The authors appear to have a creditable background and research experience which gives the article credibility and allows for an interesting insight into student mental well-being within Nepal. The paper proposes that female students are are at greater risk of reduced self-esteem and the causality needs now to be ascertained in order reduce further stigma and shame. Comparisons have been made with relevant contemporary research and this allows for a global overview of the topic and how this article fits within the body of evidence.

 I think that the article is correct not to generalize and to highlight the subjective nature of assessing se-esteem but no doubt the data will highlight the need to support and the participants may have developed a collective consciousness on the necessity to self-care in order to be effective healthcare practitioners. 

 I wish the authors well with their future efforts to support this cohort and in progressing future research and support networks which I am sure will provide long-term benefits to the health and well-being of medical students with results that can be referred to the wider student healthcare field.

Is the work clearly and accurately presented and does it cite the current literature?

Yes

If applicable, is the statistical analysis and its interpretation appropriate?

Yes

Are all the source data underlying the results available to ensure full reproducibility?

Yes

Is the study design appropriate and is the work technically sound?

Yes

Are the conclusions drawn adequately supported by the results?

Yes

Are sufficient details of methods and analysis provided to allow replication by others?

Yes

Reviewer Expertise:

I am a qualified researcher and mental health specialist with experience as an educator within higher education in undergraduate and postgraduate programmes across nursing, medical and health and social care provision.

I confirm that I have read this submission and believe that I have an appropriate level of expertise to confirm that it is of an acceptable scientific standard.
